# Field‐based individual plant phenotyping of herbaceous species by unmanned aerial vehicle

**DOI:** 10.1002/ece3.6861

**Published:** 2020-10-19

**Authors:** Wei Guo, Yuya Fukano, Koji Noshita, Seishi Ninomiya

**Affiliations:** ^1^ Institute for Sustainable Agro‐Ecosystem Services Graduate School of Agricultural and Life Sciences The University of Tokyo Tokyo Japan; ^2^ Department of Biology Kyushu University Fukuoka Japan; ^3^ Japan Science and Technology Agency Precursory Research for Embryonic Science and Technology Saitama Japan; ^4^ Plant Phenomics Research Center Nanjing Agricultural University Nanjing China

**Keywords:** image analysis, individual plant segmentation, plant phenotyping

## Abstract

1. Recent advances in Unmanned Aerial Vehicle (UAVs) and image processing have made high‐throughput field phenotyping possible at plot/canopy level in the mass grown experiment. Such techniques are now expected to be used for individual level phenotyping in the single grown experiment.
2. We found two main challenges of phenotyping individual plants in the single grown experiment: plant segmentation from weedy backgrounds and the estimation of complex traits that are difficult to measure manually.
3. In this study, we proposed a methodological framework for field‐based individual plant phenotyping by UAV. Two contributions, which are weed elimination for individual plant segmentation, and complex traits (volume and outline) extraction, have been developed. The framework demonstrated its utility in the phenotyping of *Helianthus tuberosus* (Jerusalem artichoke), an herbaceous perennial plant species.
4. The proposed framework can be applied to either small and large scale phenotyping experiments.

## Graphical abstract

A framework for field‐based individual plant phenotyping by UAV has been developed. Two main contributions, which are weed elimination for individual plant segmentation, and complex traits (volume and outline) extraction for Jerusalem artichoke, have been demonstrated in the paper.

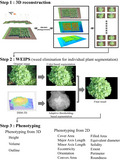

## 1 INTRODUCTION

Plant phenotyping involves the comprehensive measurement of the physical and biochemical traits of plant genotypes under specific environmental conditions and provides essential information for the plant sciences. Recent advances in technical and analytical methods have made high‐throughput field phenotyping possible (Furbank & Tester, 2011; Houle et al., 2010; Tardieu et al., 2017; Tripodi et al., 2018). Proximal sensing through the use of unmanned aerial vehicles (UAVs) is among the most promising and popular techniques for field phenotyping owing to its rapidity, nondestructiveness, cost‐effectiveness, and information density (Chapman et al., 2014; Maes & Steppe, 2019; Sankaran et al., 2015; Yang et al., 2017). UAV sensing platforms developed for agriculture also can used in genetics, ecology, forestry, and environmental science (Carrasco‐Escobar et al., 2019; Christie et al., 2016; Zhang et al., 2016). However, further methodological development is necessary for their use to become common in other fields of plant science (Minervini et al., 2015; Roth et al., 2018).

One crucial technique that remains to be addressed is the development of individual plant phenotyping (IPP). Field experiments with individually grown plants are ubiquitous in basic and applied plant science, such as typical garden experiments in ecological and evolutionary studies, tree breeding, vegetable cultivation. To date, UAV sensing has focused mainly on the measurement of plant traits as the values of group or plot units of mass grown plants, such as wheat, rice, and maize. On the other hand, little efforts have been made toward UAV sensing for traits of individually grown plants. Individual data provided by IPP will broaden the application of UAV‐based phenotyping for several reasons. First, data on individual plants allow us to examine variations within a group or plot. Whereas measurement in a group or plot unit usually provides an average trait value, IPP provides the traits of all individuals and can show trait variations. Compared with genetically uniform crops, wild plants show large genetic variation among individuals, so it is essential to capture the individual variations in phenotyping studies of wild plants or genetically diverse crops. Second, IPP might be able to test plant‐plant interactions in field conditions. Plants can influence each other either negatively (through competition) or positively (through facilitation). By monitoring temporal changes in individual growth by UAV‐based IPP, we might capture the large‐scale dynamics of plant interactions in field conditions. Thirdly, by combining with local environmental data collected by field Internet of things (IOT) devices, UAV‐based IPP can be a novel tool to examine fine‐scale genotype‐environment interactions of individual plants in the field. However, despite these great potential contributions of UAV‐based IPP to plant research, few attempts have been made to develop IPP, except for several studies of individual tree phenotyping (mostly focusing on tree height) (Díaz‐Varela et al., 2015; Fujimoto et al., 2019; Mu et al., 2018; Zarco‐Tejada et al., 2014).

One of the challenges for IPP under field conditions is the segmentation of individual plants from weedy backgrounds in image analysis. For instance, the experiment that grows single plants at a relatively low density in the field can promote the germination and growth of weeds. Even those small and low‐density weeds are not to impede the development of the focal species, because their textural and reflectance properties are often similar, it becomes a significant technical problem when segmenting the boundaries of each plant of the target species from the image. To mention, it is also not realistic to remove all weeds in a large‐scale field experiment manually. Therefore, to develop UAV‐based IPP, it is necessary to devise a technique to segment each plant of the target species, even among weeds.

Here, we present a methodological framework for UAV‐based IPP and demonstrate its utility in the phenotyping of *Helianthus tuberosus* L. (Jerusalem artichoke), an herbaceous perennial plant species. First, we developed a WEIPS (weed elimination for individual plant segmentation) method to segment each plant of the focal species in images with weeds. To evaluate its reliability, we compared areas of individual plants segmented by WEIPS with those delineated manually. Second, we tested the versatility of our framework by comparing individual plant heights estimated from images taken with those measured by hand. Finally, we illustrate the broader application of our framework by showing that it detects significant phenotypic variations among source populations of *Helianthus tuberosus* in various traits that are difficult to measure manually and requires extensive labors, such as height, volume, and outline.

## 2 MATERIALS AND METHODS

### 2.1 Growth and measurement of *Helianthus tuberosus*

*Helianthus tuberosus* L. (Jerusalem artichoke) is native to North America (Swanton et al., 1992). Because it produces large quantities of edible tubers, *H. tuberosus* was an essential crop for native North Americans before European contact (Kays & Nottingham, 2007). The species has only been weakly domesticated, so high levels of genetic diversity exist among individuals and populations in physiological, morphological, and life‐history traits (Kays & Kultur, 2005; Puttha et al., 2012; Swanton et al., 1992). Also, it has become naturalized and invasive in many regions of the world (Tesio et al., 2012; Weber & Gut, 2004).

We purchased seed tubers of *H. tuberosus* from three private farms in Tochigi, Chiba, and Gunma prefectures, Japan. Because these farms are at least 80 km apart from each other, we treated the plants from each farm as a distinct population. The seed tubers were divided and planted into individual nursery pots (6 cm in diameter; 0.3 L volume) in a commercial soil mixture (Golden; Iris Ohyama Co.). Total sixty germinated sprouts were transplanted in random order 1 m apart into three‐row plots in a crop field at the Institute for Sustainable Agro‐Ecosystem Services of the University of Tokyo (35°44′03″N, 139°32′22″E) on 28 April 2017. The rows were covered with plastic mulch film (60 cm width). Because this experiment had different research purposes, some plants were paired or grouped, but these plants did not affect the growth of focal individuals grown singly and were omitted from the subsequent analyses. For more details, see our previous study (Fukano et al., 2019). We measured individual height and stem diameter five times during plant development (13 May, 1, 13, 30 June, and 14 July) by using ruler and caliper, respectively.

### 2.2 Imaging by UAV

A low‐cost commercial UAV (DJI Inspire 1, DJI) with a built‐in camera (Zenmuse X5 Pro; 17.3 mm × 13.0 mm CMOS, 4,608 × 3,456 pixels; 16 MB with JPEG format) was flown over the field along a predesigned waypoint mission controlled by a commercial mobile phone application (Litch; VC Technology Ltd.). The waypoint mission plotted a double‐grid at an altitude of 15 m, a cruising speed of 2.5 m/s, camera looking downward, a >90% overlap of photographs to the front and sides, and an average ground sampling distance (GSD) of ~4 mm/pixel. Six ground control points (GCPs) made by acrylic plates were placed evenly (four corners and two nearly central) on the field and measured by Hemisphere RTK differential GNSS devices (Hemisphere GNSS). The point cloud was georeferenced by using a combination of direct georeferencing and the six GCPs. The mean root mean square of each computed GCP was 8.5 mm in the *x*‐direction, 16.4 mm in the *y*‐direction, and 20.1 mm in the *z*‐direction. UAV flight campaigns were conducted nine times during plant development (16, 31 May, 4, 12, 16, 29 June, 3, 7, 10 July).

### 2.3 Three‐dimensional reconstruction and plot segmentation

**FIGURE 1:**
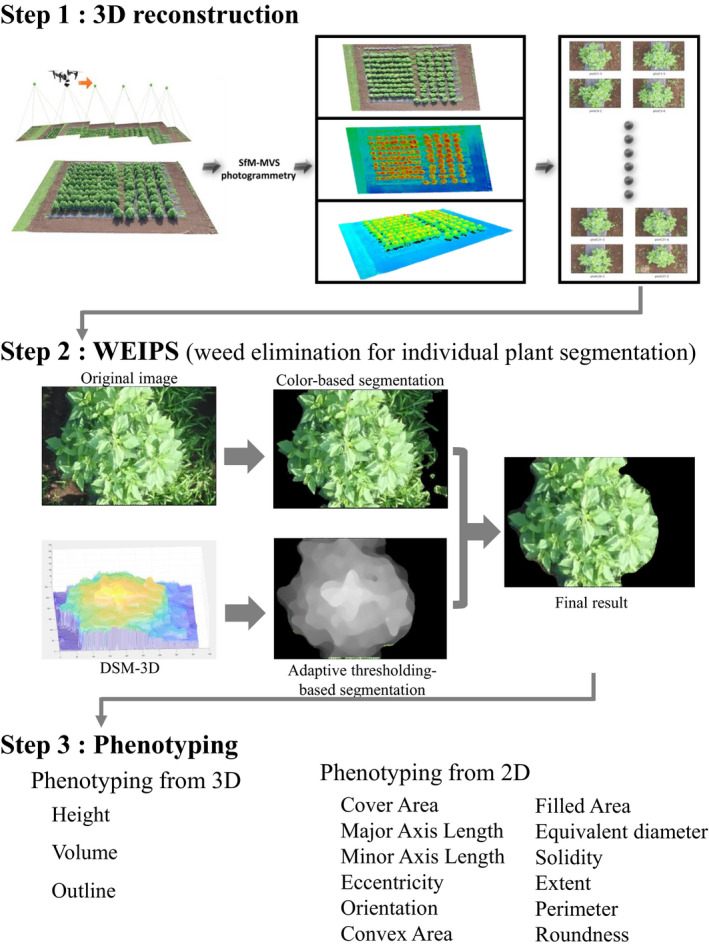
The whole process for field‐based individual plant phenotyping by UAV. Step 1: Imaging by UAV, three‐dimensional reconstruction, and plot segmentation. Step 2: Weed elimination for individual plant segmentation (WEIPS). Step 3: Individual plant phenotyping

From a set of multi‐view two‐dimensional images, canopy architectures were reconstructed as point cloud data by Structure from Motion (SfM) and Multi‐View Stereo (MVS) algorithms in Pix4Dmapper software (Pix4D SA). SfM is a photogrammetric technique used to simultaneously estimate the depth of corresponding points and camera position and direction from a set of multi‐view images. First, corresponding points among images are detected based on local features (e.g., SIFT, ORB, AKAZE; see (Tareen & Saleem, 2018) for details). Second, both extrinsic camera parameters (i.e., position and orientation) of a pair of views and the depth of corresponding points are estimated simultaneously by solving linear equations of the relation between camera coordinate frames calibrated with intrinsic camera parameters (e.g., focal length and principal point). A sparse point cloud is obtained as a set of corresponding points in three‐dimensional space by successively applying this estimation procedure to arbitrary pairs of multi‐view images. Third, several camera parameters and the point cloud are refined through bundle adjustment, which is a iterative optimization method to solve nonlinear equation, here, subject to minimization of reprojection error, see (Hartley & Zisserman, 2004) for details. Finally, MVS generates a dense point cloud based on the set of multi‐view images and camera parameters estimated in SfM. In our analysis, we used default intrinsic parameters provided by Pix4Dmapper as initial values. An orthomosaic image of the whole field was then generated from the Digital Surface Model (DSM) based on the dense point cloud (Figure 1, step 1).

We then extract the plot images manually. First, by using the “fishnet” function of ArcGIS 10.5 software (ESRI), a net of adjacent rectangular cells are generated according to user‐input numbers of rows and columns inside the predefined field boundary. Then, the plot ID is semiautomatically recorded in an attribute table by the Field Calculator tool of ArcGIS. Finally, a shapefile that contains all plots information is exported to a self‐developed Matlab (MATLAB v. R2017b, MathWorks Inc.) script to extract corresponded plot images from both the DSM and the orthomosaic.

### 2.4 WEIPS

The image segmentation process is needed to extract individual plants from plot images. In most cases, images are segmented by color on account of a large contrast between plants and bare soil (Fan et al., 2018; Guo et al., 2013, 2017). However, if the background includes objects with similar colors, such as weeds, further processing is needed. Therefore, researchers proposed several methods to distinguish weeds from the plant. For example, the use of a specific camera that can provide more spectral information; the use of complex algorithms such as machine learning with the manual selection of features and models, deep learning with the manual labeled training data (Chandra et al., 2020; Guo et al., 2018; Pérez‐Ortiz et al., 2015; Sa et al., 2017, 2018; Yu et al., 2019). Here, relying on the height of *H. tuberosus*, we propose the simple WEIPS method to segment plants from weeds. The core conception of WEIPS is segmentation by both color and height (Figure 1, step 2). In parallel, the segmented plot images from orthomosaic are processed by a machine‐learning‐based color pixel segmentation method, and the segmented plot images from DSM are processed by adaptive thresholding of height. Both methods extract a fixed polygon mask that indicates the candidate region. The masks are combined to render an individual plant without weeds.

### 2.5 Individual plant phenotyping

Several phenotypic traits are extracted from the segmented individual plants. The cover area, major and minor axis lengths, eccentricity, orientation, convex area, filled area, equivalent diameter, solidity, extent, perimeter, and roundness can be easily calculated by MATLAB function “regionprops” (Figure 1, step 3). The height, volume, and outline are computed from the corresponded segmented DSM as following algorithms.

#### 2.5.1 Height

Plant height is calculated as the difference between the plant boundary and the ground level subtracted from the DSM (Hu et al., 2018; Watanabe et al., 2017). We used ground elevations generated from the first flight as the reference (*E*_r_). For each flight, the 99th percentile of DSM value of the plant region is extracted according to the result of WEIPS (*E*_p_ *_i_*). The plant height for the *i*th flight (*H*_p_ *_i_*) is defined as:

$$H_{pi}=E_{pi}‐E_{r}$$

#### 2.5.2 Volume

Plant volume is approximated as the sum of height × area of all pixels at the plant base:

$$\text{Vol}\approx \sum_{i=1}^{n}f\left(\xi_{i},\eta_{i}\right)\Delta \sigma_{i}$$

where $f\left(\xi_{i},\eta_{i}\right)$ is the height of approximated cylinder cube, Δ*σ_i_* is the area of pixel *i*, and *n* = the number of plant pixels acquired by WEIPS.

#### 2.5.3 Outline

**FIGURE 2:**
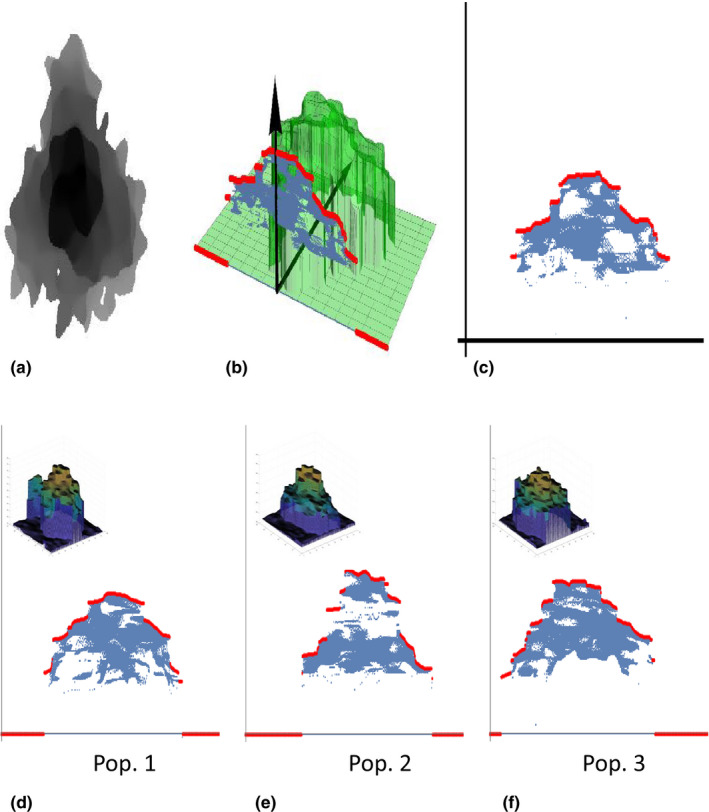
Modeling of outlines and variation among source populations. (a) The top view of DSM for an individual plant. (b) Green surface, blue dots, and red dots represent original DSM, projected DSM along a ridge, and the canopy outline as the upper boundary of the projected DSM. (c) The projected DSM on the 2D plane which perpendicular to the ridge. (d–f) Representative outline forms of three cultivars (C, G, T)

We defined the canopy outline as the upper boundary of the projected DSM along a planting line (Figure 2b,c). Here, the upper boundary is represented as a vector in which elements are the 99th percentile of height values of projected points derived from the DSM. The bin width of each element was 0.375 cm and the number of elements (i.e., size of vector) was 433. The dissimilarity between arbitrary two outlines is calculated as the minimum distance between two vectors in which both sliding and horizontal flipping are allowed. In other words, we compared among canopy outlines in terms of form, which is the geometric invariant of both translation and rotation. Here, *n*‐sliding was defined as an operation to append *n* elements of 0 to the head of the vector and delete tail *n* elements when *n* is less than 0 and to append *n* elements of 0 to the tail and delete *n* head elements when *n* is greater than 0. Horizontal mirror‐flip is defined as an operation to reverse the order of elements. In this study, we calculated the dissimilarity among all pairs of outlines with allowing up to 108‐sliding and horizontal flipping.

### 2.6 Statistical analyses

To validate the WEIPS method, we examined the correlation between the canopy coverage rate and the height of individual plants segmented by WEIPS and those manually segmented by author YF, using Pearson's correlation analysis, for each of five measurement dates.

To illustrate the utility of our framework in‐field phenotyping, we examined the phenotypic variations among the three *H. tuberosus* populations in various traits estimated from UAV imaging (Figure 1) and measured manually (height and stem diameter). The trait values were treated as response variables, and the source populations and planting row were treated as explanatory variables. All trait values were fitted to generalized linear models with a Gaussian distribution. Likelihood ratio tests were used to evaluate the significance of the explanatory variables, excluding the outline. For outline, permutational multivariate analysis of variance (PERMANOVA; Anderson, 2001; Mcardle et al., 2001) was conducted to evaluate the effect among a population by using the pairwise Adonis package (Martinez, 2020) in the R language (R Core Team, 2020) because only dissimilarities could be calculated.

## 3 RESULTS

### 3.1 Development of framework for UAV‐based IPP

We developed an easy‐to‐use framework for UAV‐based IPP (Figure 1) that requires only a commercial‐level UAV as hardware, and all processes are easy to implement.

### 3.2 Comparison between WEIPS method and manual segmentation

We developed a novel technique, WEIPS, to eliminate weeds from UAV images by combining color‐based segmentation and adaptive thresholding‐based segmentation. The performance of WEIPS has been evaluated by Qseg, which is a well‐known measurements of vegetation segmentation method (Guo et al., 2013; Meyer & Neto, 2008). Qseg is defined as below:

$$Q\text{seg}=\frac{\sum_{i=0}^{i=m}\sum_{j=0}^{j=n}\left(A{\left(v\right)}_{i,j}\cap B{\left(v\right)}_{i,j}\right)}{\sum_{i=0}^{i=m}\sum_{j=0}^{j=n}\left(A{\left(v\right)}_{i,j}\cup B{\left(v\right)}_{i,j}\right)}$$

where A is the set of the vegetation pixels (*v* = 255) or background pixels (*v* = 0) identified by a classification model, B is a reference set of manually segmented vegetation pixels (*v* = 255) or background pixels (*v* = 0), m and n are the image row and column sizes, and *i*, *j* are the pixel coordinate indices of the images. The more consistent pixels between A and B, the values become the larger ranging from 0 to 1. Namely, the higher the value, the more accurate the segmentation is.

**FIGURE 3:**
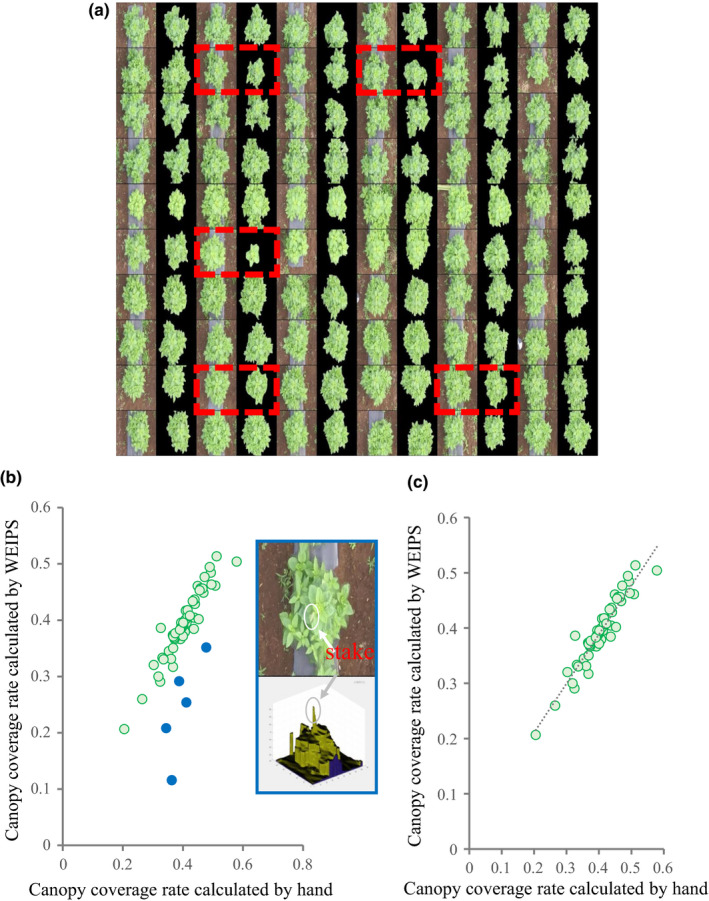
Performance of WEIPS. (a) The final segmentation of all individuals by WEIPS. Each individual is shown as a pair of images, the images on the left (with background) are the original images cropped from orthomosaic, the images on the right are the extracted individual plants. Only five plants (boxed with dashed red lines) were poorly segmented. (b) Correlation between the canopy coverage rate of individual plants segmented by WEIPS and that of plants segmented by hand. Blue dots mark the five exceptions. (c) Correlation between the canopy coverage rate of individual plants segmented by WEIPS and that of plants segmented by hand after removal of the five exceptions

The WEIPS method correctly (Qseg = 0.90) separated the weeds from the images and segmented the individual plants of the focal species, except for five individuals (surrounded by a dotted line; Figure 3a). We found a significant but relatively weak correlation between the canopy cover area of individual plants segmented by WEIPS and those manually segmented (*R* = 0.7843, Figure 3b). The correlation was weakened by the five exceptions (Figure 3b, blue points). A careful check of the photographs, DSM data, and WEIPS results revealed that the failure to segment those five exceptions were due to the stakes used to support the plants. In three‐dimensional reconstruction, the point cloud data of the five plants included the structure of the stakes, which caused the overestimation of plant height and resulted in an inadequate threshold for the segmentation. When we excluded the five individuals from the analysis, the correlation between WEIPS and manual segmentation became stronger (*R* = 0.9472, Figure 3c).

### 3.3 Comparison between estimated and measured heights

**FIGURE 4:**
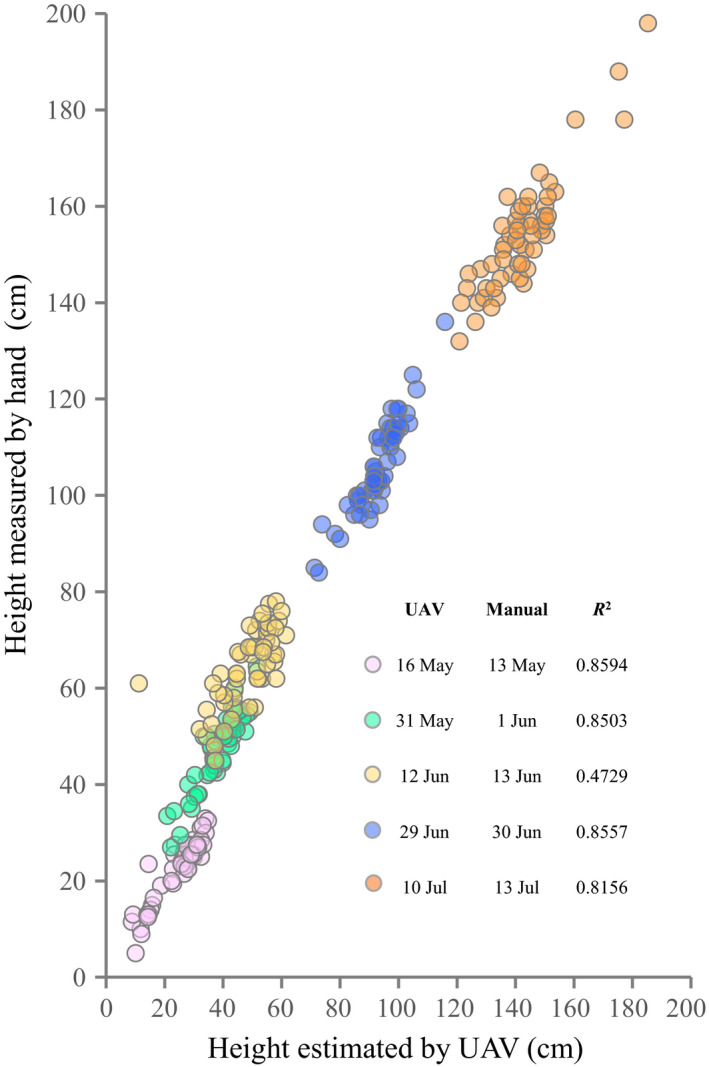
Correlation between plant height estimated from the UAV images and that measured by hand on all measurement dates

There were significant correlations between plant height estimated from the UAV images and that measured by hand on all measurement dates (*p* < .001 for all dates, Figure 4). The correlations were relatively high (*R* ^2^ ≈ 0.85) except on 12 June (*R* ^2^ = 0.47).

### 3.4 Phenotypic variation among source populations

The results of all statistical tests are shown in Tables S1 and S2. We detected significant variations among the source populations in several traits estimated by UAV‐based IPP. For example, for the data obtained from 16 May, minor axis length, height, and volume differed significantly among populations (*p* = .0053, <.001, <.001, respectively). Other traits did not differ among populations. The outline differed between population 1 and 2 and population 1 and 3 (*p* = .014 and <.001, respectively) but not differed between population 1 and 2 (*p* = .084).

## 4 DISCUSSION

We propose a new methodological framework for UAV‐based IPP. Overall, it successfully captured a range of individual traits, including height, volume, cover area, and outline, of field‐grown *H. tuberosus* in the presence of weeds. The WEIPS method achieved high‐accuracy segmentation of the focal plants from images with weeds (Figure 3c). The estimated individual plant heights showed consistently high correlation with the measured heights except on one of five measurement days (Figure 4). The framework detected temporal changes in phenotypic variations among populations of *H. tuberosus* in various traits that are difficult to measure manually and require extensive labors, such as height, volume, and outline (Figure 2). We believe that this noninvasive, cost‐effective, labor‐saving framework can become a standard method for individual phenotyping of field‐grown plants.

The framework will shed new light on and improve research efficiency in both basic and applied plant biology. Its most remarkable feature is that it can estimate several shape‐related traits that are difficult to measure manually (e.g., outline of aboveground parts and roundness). The ecological and evolutionary relevance of individual plant shape has received relatively little attention, probably owing to difficulties in noninvasive measurement. By using this framework, we might be able to examine which ecological and evolutionary factors influence the aboveground plant shape in field conditions. The framework can be easily applied to phenotyping in typical garden experiments, which is a classical approach to quantifying genetically based phenotypic differentiation among populations (Colautti et al., 2009). Because the UAV‐based IPP saves labor, the use of the framework will improve research efficiency significantly. Recent studies have developed methods for automatically detecting crop head/flowering in time‐series RGB images (Desai et al., 2019; Ghosal et al., 2019; Guo et al., 2018). By combining these methods and UAV‐based IPP, we can quantify the dynamics and interactions between aboveground morphological traits and phenological traits under field conditions.

In the applied plant sciences, our framework is handy for large‐scale and long‐term phenotyping of vegetable species and tree seedlings, which are grown individually. Large‐scale individual phenotyping will accelerate the breeding process, especially in genetically diverse crops such as *H. tuberosus*. Furthermore, by combining environmental data sensed by the field IOT platform, we can examine fine‐scale genotype–environment interactions and learn how micro‐environmental variations affect the growth and yield of individual crop plants.

While our results demonstrate the utility of our framework for UAV‐based IPP, they also demonstrate the limitations in fieldwork. First, plant stakes caused the overestimation of plant height, compromising the threshold level for segmentation. Thus, for UAV‐based phenotyping, we suggest removing other artifacts as well as GCPs from the imagery. Second, on one of five days on which plant height was measured, the correlation between the height estimated by the proposed framework and the height measured manually was low. Although we could not determine the reason, microclimate conditions such as wind disturbance might reduce the resolution of the point cloud (Table S3). Third, the proposed WEIPS relays on the height difference between plants and weeds. If the weeds (or another plant) are of the same height to the plant and overlapped, it will not work correctly. To further extend this framework to phenotyping of other types of plants, we need to handle these limitations.

## CONFLICT OF INTEREST

The authors declare no conflict of interest.

## AUTHOR CONTRIBUTION

**Wei Guo:** Conceptualization (equal); Data curation (lead); Formal analysis (lead); Funding acquisition (lead); Investigation (lead); Methodology (lead); Project administration (lead); Resources (lead); Software (lead); Supervision (lead); Validation (lead); Visualization (lead); Writing‐original draft (lead); Writing‐review & editing (lead). **Yuya Fukano:** Conceptualization (equal); Data curation (equal); Formal analysis (equal); Funding acquisition (equal); Investigation (equal); Methodology (equal); Project administration (equal); Resources (equal); Supervision (equal); Validation (equal); Visualization (equal); Writing‐original draft (equal); Writing‐review & editing (equal). **Koji Noshita:** Conceptualization (equal); Data curation (equal); Formal analysis (equal); Methodology (equal); Validation (supporting); Visualization (supporting); Writing‐original draft (supporting); Writing‐review & editing (supporting). **Seishi Ninomiya:** Conceptualization (supporting); Funding acquisition (equal); Project administration (equal); Supervision (supporting); Writing‐original draft (supporting); Writing‐review & editing (supporting).

### OPEN RESEARCH BADGES

This article has earned an Open Data Badge for making publicly available the digitally‐shareable data necessary to reproduce the reported results. The data is available at https://doi.org/10.5061/dryad.0cfxpnw0b

## Supporting information

Table S1

Table S2

Table S3

## Data Availability

Supportive data and source code are available at the support page is here as follows: https://doi.org/10.5061/dryad.0cfxpnw0b

## References

[ece36861-bib-0001] Anderson, M. J. (2001). A new method for non‐parametric multivariate analysis of variance. Austral Ecology, 26, 32–46.

[ece36861-bib-0002] Carrasco‐Escobar, G. , Manrique, E. , Ruiz‐Cabrejos, J. , Saavedra, M. , Alava, F. , Bickersmith, S. , Prussing, C. , Vinetz, J. M. , Conn, J. E. , Moreno, M. , & Gamboa, D. (2019). High‐accuracy detection of malaria vector larval habitats using drone‐based multispectral imagery. PLoS Neglected Tropical Diseases, 13(1), 1–24. 10.1371/journal.pntd.0007105 PMC6353212 30653491

[ece36861-bib-0003] Chandra, A. L. , Desai, S. V. , Balasubramanian, V. N. , Ninomiya, S. , & Guo, W. (2020). Active learning with weak supervision for cost‐effective panicle detection in cereal crops. Plant Methods, 16, 34 10.1186/s13007-020-00575-8 32161624 PMC7060654

[ece36861-bib-0004] Chapman, S. , Merz, T. , Chan, A. , Jackway, P. , Hrabar, S. , Dreccer, M. , Holland, E. , Zheng, B. , Ling, T. , & Jimenez‐Berni, J. (2014). Pheno‐copter: A low‐altitude, autonomous remote‐sensing robotic helicopter for high‐throughput field‐based phenotyping. Agronomy, 4(2), 279–301. 10.3390/agronomy4020279

[ece36861-bib-0005] Christie, K. S. , Gilbert, S. L. , Brown, C. L. , Hatfield, M. , & Hanson, L. (2016). Unmanned aircraft systems in wildlife research: Current and future applications of a transformative technology. Frontiers in Ecology and the Environment, 14(5), 241–251. 10.1002/fee.1281

[ece36861-bib-0006] Colautti, R. I. , Maron, J. L. , & Barrett, S. C. H. (2009). Common garden comparisons of native and introduced plant populations: Latitudinal clines can obscure evolutionary inferences. Evolutionary Applications, 2(2), 187–199. 10.1111/j.1752-4571.2008.00053.x 25567860 PMC3352372

[ece36861-bib-0007] Desai, S. V. , Balasubramanian, V. N. , Fukatsu, T. , Ninomiya, S. , & Guo, W. (2019). Automatic estimation of heading date of paddy rice using deep learning. Plant Methods, 15(1), 76 10.1186/s13007-019-0457-1 31338116 PMC6626381

[ece36861-bib-0008] Díaz‐Varela, R. A. , de la Rosa, R. , León, L. , & Zarco‐Tejada, P. J. (2015). High‐resolution airborne UAV imagery to assess olive tree crown parameters using 3D photo reconstruction: Application in breeding trials. Remote Sensing, 7(4), 4213–4232. 10.3390/rs70404213

[ece36861-bib-0009] Fan, X. , Kawamura, K. , Guo, W. , Xuan, T. D. , Lim, J. , Yuba, N. , Kurokawa, Y. , Obitsu, T. , Lv, R. , Tsumiyama, Y. , Yasuda, T. , & Wang, Z. (2018). A simple visible and near‐infrared (V‐NIR) camera system for monitoring the leaf area index and growth stage of Italian ryegrass. Computers and Electronics in Agriculture, 144, 314–323. 10.1016/j.compag.2017.11.025

[ece36861-bib-0010] Fujimoto, A. , Haga, C. , Matsui, T. , Machimura, T. , Hayashi, K. , Sugita, S. , & Takagi, H. (2019). An end to end process development for UAV‐SfM based forest monitoring: Individual tree detection, species classification and carbon dynamics simulation. Forests, 10(8), 1–27. 10.3390/f10080680

[ece36861-bib-0011] Fukano, Y. , Guo, W. , Noshita, K. , Hashida, S. , & Kamikawa, S. (2019). Genotype‐aggregated planting improves yield in Jerusalem artichoke (*Helianthus tuberosus*) due to self/non‐self‐discrimination. Evolutionary Applications, 12(3), 508–518. 30828371 10.1111/eva.12735 PMC6383731

[ece36861-bib-0012] Furbank, R. T. , & Tester, M. (2011). Phenomics – technologies to relieve the phenotyping bottleneck. Trends in Plant Science, 16(12), 635–644. 10.1016/j.tplants.2011.09.005 22074787

[ece36861-bib-0013] Ghosal, S. , Zheng, B. , Chapman, S. C. , Potgieter, A. B. , Jordan, D. R. , Wang, X. , Singh, A. K. , Singh, A. , Hirafuji, M. , Ninomiya, S. , Ganapathysubramanian, B. , Sarkar, S. , & Guo, W. (2019). A weakly supervised deep learning framework for sorghum head detection and counting. Plant Phenomics, 2019, 1–14. 10.34133/2019/1525874 PMC7706102 33313521

[ece36861-bib-0014] Guo, W. , Rage, U. K. , & Ninomiya, S. (2013). Illumination invariant segmentation of vegetation for time series wheat images based on decision tree model. Computers and Electronics in Agriculture, 96, 58–66. 10.1016/j.compag.2013.04.010

[ece36861-bib-0015] Guo, W. , Zheng, B. , Duan, T. , Fukatsu, T. , Chapman, S. , & Ninomiya, S. (2017). EasyPCC: Benchmark datasets and tools for high‐throughput measurement of the plant canopy coverage ratio under field conditions. Sensors (Switzerland), 17(4), 1–13. 10.3390/s17040798 PMC5422159 28387746

[ece36861-bib-0016] Guo, W. , Zheng, B. , Potgieter, A. B. , Diot, J. , Watanabe, K. , Noshita, K. , Jordan, D. R. , Wang, X. , Watson, J. , Ninomiya, S. , & Chapman, S. C. (2018). Aerial imagery analysis – Quantifying appearance and number of sorghum heads for applications in breeding and agronomy. Frontiers in Plant Science, 9, 1–9. 10.3389/fpls.2018.01544 30405675 PMC6206408

[ece36861-bib-0017] Hartley, R. , & Zisserman, A. (2004). Multiple view geometry in computer vision (2nd ed.) Cambridge University Press 10.1017/CBO9780511811685

[ece36861-bib-0018] Houle, D. , Govindaraju, D. R. , & Omholt, S. (2010). Phenomics: The next challenge. Nature Reviews Genetics, 11(12), 855–866. 10.1038/nrg2897 21085204

[ece36861-bib-0019] Hu, P. , Chapman, S. C. , Wang, X. , Potgieter, A. , Duan, T. , Jordan, D. , Guo, Y. , & Zheng, B. (2018). Estimation of plant height using a high throughput phenotyping platform based on unmanned aerial vehicle and self‐calibration: Example for sorghum breeding. European Journal of Agronomy, 95, 24–32. 10.1016/j.eja.2018.02.004

[ece36861-bib-0020] Kays, S. J. , & Kultur, F. (2005). Genetic variation in Jerusalem artichoke (*Helianthus tuberosus* L.) flowering date and duration. HortScience, 40(6), 1675–1678. 10.21273/HORTSCI.40.6.1675

[ece36861-bib-0021] Kays, S. J. , & Nottingham, S. F. (2007). Biology and chemistry of Jerusalem Artichoke: *Helianthus tuberosus* L. CRC Press 10.1201/9781420044966

[ece36861-bib-0022] Maes, W. H. , & Steppe, K. (2019). Perspectives for remote sensing with unmanned aerial vehicles in precision agriculture. Trends in Plant Science, 24(2), 152–164. 10.1016/j.tplants.2018.11.007 30558964

[ece36861-bib-0023] Martinez, Arbizu, P. (2020). pairwiseAdonis: Pairwise multilevel comparison using adonis. R Packag. Version 0.4.. Available online: https://github.com/pmartinezarbizu/pairwiseAdonis (accessed on 26 September 2020 ).

[ece36861-bib-0024] Mcardle, B. H. , Anderson, M. J. , Ecology, S. , & Jan, N. (2001). Fitting multivariate models to community data. Ecological Society of America: Issues in Ecology, 82(1), 290–297. https://esajournals.onlinelibrary.wiley.com/doi/full/10.1890/0012‐9658%282001%29082%5B0290%3AFMMTCD%5D2.0.CO%3B2

[ece36861-bib-0025] Meyer, G. E. , & Neto, J. C. (2008). Verification of color vegetation indices for automated crop imaging applications. Computers and Electronics in Agriculture, 63(2), 282–293. 10.1016/j.compag.2008.03.009

[ece36861-bib-0026] Minervini, M. , Scharr, H. , & Tsaftaris, S. A. (2015). Image analysis: The new bottleneck in plant phenotyping [Applications Corner]. IEEE Signal Processing Magazine, 32(4), 126–131. 10.1109/MSP.2015.2405111

[ece36861-bib-0027] Mu, Y. , Fujii, Y. , Takata, D. , Zheng, B. , Noshita, K. , Honda, K. , Ninomiya, S. , & Guo, W. (2018). Characterization of peach tree crown by using high‐resolution images from an unmanned aerial vehicle. Horticculture Research, 5, 74 10.1038/s41438-018-0097-z PMC6286954 30564372

[ece36861-bib-0028] Pérez‐Ortiz, M. , Peña, J. M. , Gutiérrez, P. A. , Torres‐Sánchez, J. , Hervás‐Martínez, C. , & López‐Granados, F. (2015). A semi‐supervised system for weed mapping in sunflower crops using unmanned aerial vehicles and a crop row detection method. Applied Soft Computing, 37, 533–544. 10.1016/j.asoc.2015.08.027

[ece36861-bib-0029] Puttha, R. , Jogloy, S. , Wangsomnuk, P. P. , Srijaranai, S. , Kesmala, T. , & Patanothai, A. (2012). Genotypic variability and genotype by environment interactions for inulin content of Jerusalem artichoke germplasm. Euphytica, 183(1), 119–131. 10.1007/s10681-011-0520-0

[ece36861-bib-0030] R Core Team (2020). R: A Language and Environment for Statistical Computing}. R Foundation for Statistical Computing https://www.R‐project.org.

[ece36861-bib-0031] Roth, L. , Hund, A. , & Aasen, H. (2018). PhenoFly planning tool: Flight planning for high‐resolution optical remote sensing with unmanned areal systems. Plant Methods, 14(1), 116 10.1186/s13007-018-0376-6 30598692 PMC6302310

[ece36861-bib-0032] Sa, I. , Chen, Z. , Popovic, M. , Khanna, R. , Liebisch, F. , Nieto, J. , & Siegwart, R. (2017). weedNet: Dense semantic weed classification using multispectral images and MAV for smart farming. IEEE Robotics and Automation Letters, 3(1), 588–595. 10.1109/LRA.2017.2774979

[ece36861-bib-0033] Sa, I. , Popović, M. , Khanna, R. , Chen, Z. , Lottes, P. , Liebisch, F. , Nieto, J. , Stachniss, C. , Walter, A. , & Siegwart, R. (2018). WeedMap: A large‐scale semantic weed mapping framework using aerial multispectral imaging and deep neural network for precision farming. Remote Sensing, 10(9), 1423 10.3390/rs10091423

[ece36861-bib-0034] Sankaran, S. , Khot, L. R. , Espinoza, C. Z. , Jarolmasjed, S. , Sathuvalli, V. R. , Vandemark, G. J. , Miklas, P. N. , Carter, A. H. , Pumphrey, M. O. , Knowles, N. R. , & Pavek, M. J. (2015). Low‐altitude, high‐resolution aerial imaging systems for row and field crop phenotyping: A review. European Journal of Agronomy, 70, 112–123. 10.1016/j.eja.2015.07.004

[ece36861-bib-0035] Swanton, C. J. , Cavers, P. B. , Clementsl, D. R. , & Moore, M. J. (1992). The biolory of Canadian weeds. 101. *Helianthus tuberosus* L. Canadian Journal of Plant Science, 72, 1367–1382.

[ece36861-bib-0036] Tardieu, F. , Cabrera‐Bosquet, L. , Pridmore, T. , & Bennett, M. (2017). Plant phenomics, from sensors to knowledge. Current Biology, 27(15), R770–R783. 10.1016/j.cub.2017.05.055 28787611

[ece36861-bib-0037] Tareen, S. A. K. , & Saleem, Z. (2018). A comparative analysis of SIFT, SURF, KAZE, AKAZE, ORB, and BRISK In 2018 International Conference on Computing, Mathematics and Engineering Technologies (iCoMET) (pp. 1–10).

[ece36861-bib-0038] Tesio, F. , Vidotto, F. , & Ferrero, A. (2012). Allelopathic persistence of *Helianthus tuberosus* L. residues in the soil. Scientia Horticulturae, 135, 98–105. 10.1016/j.scienta.2011.12.008

[ece36861-bib-0039] Tripodi, P. , Massa, D. , Venezia, A. , & Cardi, T. (2018). Sensing technologies for precision phenotyping in vegetable crops: Current status and future challenges. Agronomy, 8(4), 57 10.3390/agronomy8040057

[ece36861-bib-0040] Watanabe, K. , Guo, W. , Arai, K. , Takanashi, H. , Kajiya‐Kanegae, H. , Kobayashi, M. , Yano, K. , Tokunaga, T. , Fujiwara, T. , Tsutsumi, N. , & Iwata, H. (2017). High‐throughput phenotyping of sorghum plant height using an unmanned aerial vehicle and its application to genomic prediction modeling. Frontiers in Plant Science, 8, 421 10.3389/fpls.2017.00421 28400784 PMC5368247

[ece36861-bib-0041] Weber, E. , & Gut, D. (2004). Assessing the risk of potentially invasive plant species in central Europe. Journal for Nature Conservation, 12(3), 171–179. 10.1016/j.jnc.2004.04.002

[ece36861-bib-0042] Yang, G. , Liu, J. , Zhao, C. , Li, Z. , Huang, Y. , Yu, H. , Xu, B. O. , Yang, X. , Zhu, D. , Zhang, X. , Zhang, R. , Feng, H. , Zhao, X. , Li, Z. , Li, H. , & Yang, H. (2017). Unmanned aerial vehicle remote sensing for field‐based crop phenotyping: Current status and perspectives. Frontiers in Plant Science, 8, 1111 10.3389/fpls.2017.01111 28713402 PMC5492853

[ece36861-bib-0043] Yu, J. , Sharpe, S. M. , Schumann, A. W. , & Boyd, N. S. (2019). Deep learning for image‐based weed detection in turfgrass. European Journal of Agronomy, 104, 78–84. 10.1016/j.eja.2019.01.004

[ece36861-bib-0044] Zarco‐Tejada, P. J. , Diaz‐Varela, R. , Angileri, V. , & Loudjani, P. (2014). Tree height quantification using very high resolution imagery acquired from an unmanned aerial vehicle (UAV) and automatic 3D photo‐reconstruction methods. European Journal of Agronomy, 55, 89–99. 10.1016/j.eja.2014.01.004

[ece36861-bib-0045] Zhang, J. , Hu, J. , Lian, J. , Fan, Z. , Ouyang, X. , & Ye, W. (2016). Seeing the forest from drones: Testing the potential of lightweight drones as a tool for long‐term forest monitoring. Biological Conservation, 198, 60–69. 10.1016/j.biocon.2016.03.027

